# Neurological and Dermatological Manifestations of Tuberous Sclerosis Complex: Report from a Romanian Tertiary Hospital Cohort

**DOI:** 10.3390/jcm12206550

**Published:** 2023-10-16

**Authors:** Adriana Octaviana Dulamea, Anca Adriana Arbune, Daniela Anghel, Voicu Boscaiu, Andreea Andronesi, Gener Ismail

**Affiliations:** 1 Neurology Clinic, Fundeni Clinical Institute, 022328 Bucharest, Romania; octaviana.dulamea@umfcd.ro (A.O.D.); daniela.anghel@umfcd.ro (D.A.); 2 Department III, Dental Medicine Faculty, “Carol Davila” University of Medicine and Pharmacy, 050474 Bucharest, Romania; 3 Institute of Mathematical Statistics and Applied Mathematics “Gheorghe Mihoc-Caius Iacob”, 050711 Bucharest, Romania; 4 Nephrology Clinic, Fundeni Clinical Institute, 022328 Bucharest, Romania; andreea.andronesi@umfcd.ro (A.A.); gener.ismail@umfcd.ro (G.I.); 5 Department 3, Medicine Faculty, “Carol Davila” University of Medicine and Pharmacy, 050474 Bucharest, Romania

**Keywords:** tuberous sclerosis complex, neuroimaging, intellectual impairment, dermatological involvement, everolimus, Charlson comorbidity index

## Abstract

Tuberous sclerosis complex is a rare multisystem genetic disorder characterized by multiorgan involvement, frequently associated with intellectual impairment and epilepsy. The aim of our study was to describe the neurological and dermatological manifestations of TSC in 32 adult patients (of whom 19 were females) who attended the Neurology and Nephrology Clinics of Fundeni Clinical Institute in Romania from 2015 to 2020. Seventeen patients were diagnosed with epilepsy, nine patients had intellectual impairment, and complete neuroimaging was available for twenty-two patients. As expected, the most frequent dermatological lesions were cutaneous angiofibromas in 20 patients, but with a lower frequency than described in the current literature. Statistical analysis was performed considering the small number of patients. Cortical tubers in neuroimaging seemed to be associated with the diagnosis of epilepsy, while subependymal nodules represented a risk factor for intellectual impairment. Males showed a larger number of dermatological types of lesions, especially café -au-lait patches. Interestingly, we found a statistically significant positive association between epilepsy and the presence of cutaneous angiofibromas, as well as total dermatological involvement. Females had significantly higher Charlson comorbidity index scores, indicating a higher burden of disease. Everolimus seemed to be a well-tolerated treatment and showed promising results in controlling epileptic seizures alone in two patients. More studies, with the inclusion of a larger number of patients, are needed to confirm these results.

## 1. Introduction

Tuberous sclerosis complex (TSC) is a rare multisystem genetic disorder characterized by the involvement of a wide range of organs, including brain, skin, kidney, lung, and eye [1,2]. There is a specific dynamic between organ involvement with age-related expression and therefore the specific organ manifestations are different during the lifespan of the individual with TSC [3]. The brain, skin, and kidney represent the commonly affected organ systems in 80–90% of patients with TSC. 

The central nervous system-related findings of TSC include structural lesions of the brain such as cortical dysplasia (cortical tubers and cerebral white matter radial migration lines), subependymal nodules (SEN) and subependymal giant cell astrocytomas (SEGA), epilepsy, and TSC-associated neuropsychiatric disorders. Cortical tubers are observed in approximately 90% of TSC patients. Both types of cortical dysplasia in TSC are commonly associated with intractable epilepsy and learning difficulties. SEN and SEGA are observed in 80% of patients with TSC. SEGAs usually arise from SEN, especially near the foramen of Monro. Although benign and slow growing, they can cause serious neurologic compromise, including obstructive hydrocephalus, and may progressively calcify over time. Epilepsy is one of the most common neurologic symptoms in patients with TSC, with a reported prevalence of 62% to 93% [4]. It is also a significant cause of morbidity and mortality in patients with TSC [4,5,6]. Epilepsy usually begins during the first months of life and, in the majority of patients, before the first year. Early onset epilepsy often presents as focal seizures initially and can precede, coexist with, or evolve into infantile spasms. However, patients with TSC may present all types of focal and generalized seizures. 

Dermatological manifestations are detected in all ages and affect more than 90% of TSC patients. Facial angiofibromas often start to appear within the first 2–5 years of life and ultimately occur in approximately 75% of patients [7,8]. Hypomelanotic macules are often the earliest and most frequently reported cutaneous findings in TSC. They present as hypopigmented macules and patches of various morphologies (medium/large “ash-leaf” type, polygonal “thumbprint like”, small “confetti-like” type) and should not be confused with de-pigmented patches found in other pigmentary disorders such as vitiligo. “Shagreen patches” are connective tissue hamartomas found in approximately half of patients with TSC. Ungual fibromas usually appear later than other TSC-associated skin lesions, typically after the second decade.

There are several therapeutic options available for the treatment of seizures associated with TSC, including antiepileptic drugs (AEDs), hormonal therapy, epilepsy surgery, ketogenic diet, and vagus nerve stimulation. However, about two-thirds of the patients develop treatment refractory epilepsy, associated with an increased rate of intellectual disability and TSC-associated neuropsychiatric disorders (TAND). The discovery of mutations in the TSC1 and TSC2 genes and their association with the mechanistic target of the rapamycin (mTOR) pathway has been the basis for the development of targeted therapy in TSC patients. A large number of randomized controlled clinical trials with mTOR inhibitors have shown efficacy in the treatment of several clinical organ manifestations of TSC, most importantly in patients with subependymal giant cell astrocytoma (SEGA), seizures (treatment resistant with focal onset), and angiomyolipoma (AML), as well as cutaneous manifestations [9,10,11,12].

The main objective of this study was to characterize a sample of patients who attended the Neurology and Nephrology Clinics of Fundeni Clinical Institute in Romania from 2015 to 2020 regarding neurological and dermatological manifestations of TSC and the therapeutic approach.

## 2. Materials and Methods

This is a retrospective single center study of a population of adult patients with TSC currently treated and followed up in the Neurology and Nephrology Clinics of Fundeni Clinical Institute in Bucharest, Romania. This study was performed in line with the principles of the Declaration of Helsinki. The study was approved by the Ethics Council of Fundeni Clinical Institute (No. 7248/08.02.2023). All patients gave their informed consent for the use of their data in the present study.

The patients were primarily referred to the nephrology clinic for renal dysfunction already diagnosed and addressed by the pediatric neurologists. The patients were managed by a multidisciplinary team formed by a cardiologist, a dermatologist, an internal medicine specialist, an ophthalmologist, and an endocrinologist. The diagnosis of TSC was made using the current diagnostic criteria and recommendations available at the time [13]. All patients’ files, written and electronic, were reviewed to assess the clinical manifestations, the initial diagnostic approach, and the management of the follow-up. These included renal assessment comprising kidney ultrasound or magnetic resonance imaging (MRI); thorax, abdominal, and pelvic computed tomography (CT); cardiological examination (electrocardiogram and echocardiography); dermatological, ophthalmological, and endocrinologic examination. The neurologic assessment included cranial CT or brain MRI, electroencephalography (EEG) studies, and neuropsychological examination. Brain imaging and thorax, abdominal, and pelvic CT-scans were performed partly at the Radiology Clinic of Fundeni Clinical Institute and partly at other sites. The imaging performed at other sites was reviewed by the same team of radiology specialists that performed and interpreted the imaging of these patients from the Fundeni Clinical Institute.

Some of the patients received therapy with mTOR inhibitors in the nephrology clinic and were followed up for a period between 1 and 5 years. The following inclusion criteria were applied: age at enrollment over 18 years and definite diagnosis of TSC according to the most recent criteria.

The indications and exclusion criteria for receiving treatment with mTOR inhibitors were established in accordance with the national Romanian protocol (compensation and the national program for tuberous sclerosis treatment was introduced in 2014; the exact criteria can be found in the L01XE10 specific form, freely available online). The final decision pertained to the patient, and some did not wish to receive mTOR treatment. The patients were periodically followed up.

The inclusion criteria according to the national protocol for mTOR treatment:

- Cerebral subependymal giant cell astrocytoma (SEGA) associated with TSC in patients ≥1 year of age who do not have a surgical indication, OR at least one SEGA lesion with the maximum diameter of ≥0.5 cm on CT/MRI imaging OR the documented increase in size of the SEGA on consecutive imaging studies;
- Renal angiomyolipoma (AML) associated with TSC that has a risk of complications (based on tumor size, the presence of an aneurysm, the presence of multiple or bilateral tumors) but does not require emergency surgery, OR renal AML lesion with maximum diameter of ≥3 cm on CT/MRI imaging, OR the documented increase in size of the renal AML on consecutive imaging studies, OR renal function evaluation and conservation (glomerular filtration rate), OR blood pressure evaluation and control;
- Since 2019, treatment resistant focal epilepsy associated with TSC, with/without secondary generalized seizures in patients ≥2 years.

The exclusion criteria according to the national protocol for mTOR treatment:

- Acute neurological symptoms due to cerebral SEGA with surgical indication
- acute symptoms due to renal AML (including hemorrhage secondary to AML) with surgical indication;
- Treatment-resistant focal epilepsy from causes other than TSC;
- Allergic reaction to sirolimus or similar components.

Data were retrospectively collected for each patient from the clinical records with a focus on age, gender, intellectual impairment (IQ < 70 according to *Diagnostic and Statistical Manual of Mental Disorders, 4th Edition* criteria), epilepsy (type of epilepsy and antiepileptic drugs used), depression, neuroimaging findings (cortical tubers, subependymal hamartomas, subependymal giant cell astrocytoma, hydrocephalia, cortical atrophy, other findings), renal involvement (angiomyolipomas, renal nodules, renal cysts), pulmonary lesions (lymphangioleiomyomatosis, cysts, nodules, fibrosis, other findings), liver and spleen involvement (nodules, lipomas, cyst, hepatomegaly, other findings), cardio-vascular disorder, cutaneous findings (facial angiomyofibromas, periungual fibromas, hypomelanotic macules, shagreen patch), bone changes, abdominal lymphadenopathies, and comorbidities. Based on collected data, the Charlson comorbidity index was calculated for each patient. Missing data were addressed by telephone interview conducted by clinical experts.

Statistical analysis used software IBM-SPSS Ver. 22.0 The following statistical tests were applied: Fisher’s exact test for contingency tables; nonparametric Mann–Whitney U-test in order to compare distributions and odds ratio statistics. Considering the fact that the available sample was small, the exact value of the statistical significances was taken into account.

## 3. Results

### 3.1. General Clinical Characteristics of the Study Population

The study included 32 TSC patients (17 with and 19 without epilepsy), of whom 19 were females (59.4%). The ages of patients included in the study varied between 19 and 68 years, with a mean of 38 years. The mean age at diagnosis was 11 years, with no statistically significant differences between female and male patients. A total of 12 patients come from rural areas (37.5%) and 20 patients came from urban areas (62.5%). Only two patients underwent genetic testing, confirming TSC 2 mutation, because the genetic testing had to be paid directly by the patients. We also calculated the Charlson comorbidity index for each patient, with a mean score of 4.38 (minimum 2, maximum 10).

### 3.2. Neurological Manifestations

Intellectual impairment was found in nine patients (40.91%). Seventeen patients (77.27%) were diagnosed with epilepsy (focal seizures or focal to bilateral tonic–clonic seizures) and 50% of patients had epilepsy since the onset of the disease.

**Figure 1:**
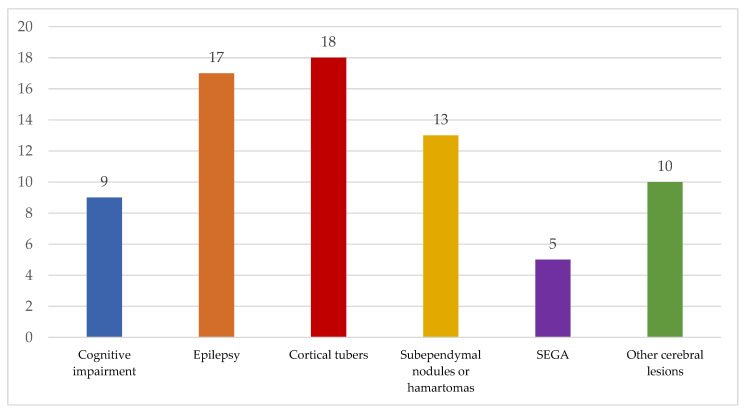
Summary of central nervous system clinical and radiological manifestations of TSC (*n* = 22).

Complete data concerning neuroimaging were found in 22 patients (Figure 1). Review of the neuroimaging of these patients showed the presence of cortical tubers in 18 patients (81.82%), subependymal nodules (SEN) in 13 patients (59.09%), and subependymal giant cell astrocytoma (SEGA) in 5 patients (22.73%). Other neuroimaging findings were observed in 10 patients (45.45%) and consisted of cortical atrophy and leukoaraiosis (one patient), discrete cerebellar oedema and lowered cerebellar tonsils (one patient), hydrocephalus (four patients), and cerebral cavernomas (one patient).

### 3.3. Dermatological Manifestations

The dermatological examination revealed cutaneous angiofibroma in 20 patients (62.5%), cutaneous angiokeratomas in 6 patients (18.8%), periungual fibromas in 7 patients (21.9%), hypomelanotic macules in 5 patients (15.6%), café-au-lait patches in 5 patients (15.6%), gingival fibromas in 4 patients (12.5%), Shagreen patches in 3 patients (9.4%), and dental pitting in 1 patient (3.1%). In our group, seven patients (21.9%) did not have any documented dermatological lesions, underlying the importance of more training of neurology and pediatric neurology specialists on the cutaneous manifestations of tuberous sclerosis. Angiofibroma seemed to be the most difficult to recognize, as the mean age at diagnosis of patients with only angiofibroma as TSC cutaneous lesions was 14 years.

### 3.4. Treatment

In our study, 11.8% (2 out of 17) patients with epilepsy did not require AEDs, obtaining a satisfactory control of seizures from treatment alone with everolimus. These patients only had cortical tubers in their neuroimaging. One patient with both brain lesions and epilepsy refused treatment with either everolimus or any AED.

**Figure 2:**
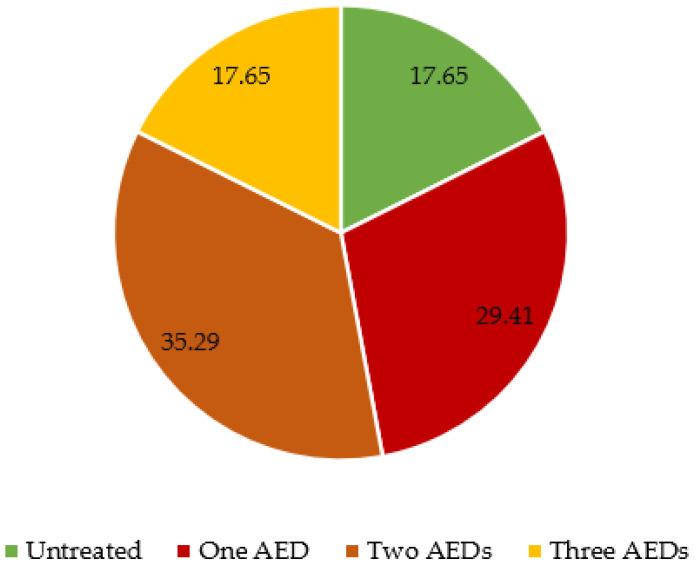
Number of needed AEDs (percentages) by patients with TSC and epilepsy (*n* = 17).

**Figure 3:**
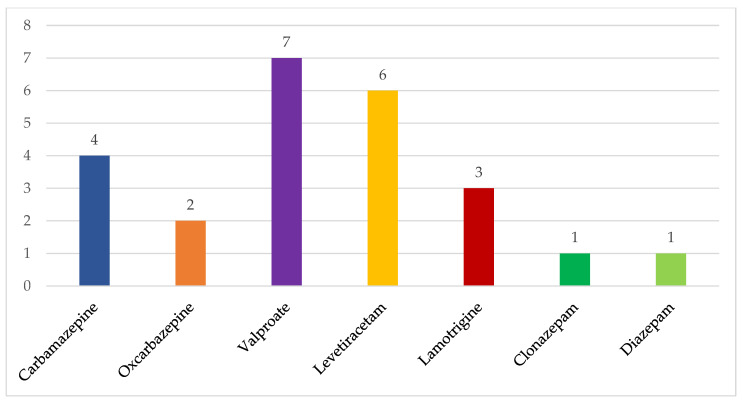
Summary of received antiepileptic drugs in TSC patients with epilepsy and tuberous sclerosis (*n* = 14).

Antiepileptic drugs (AED) were given to 14 patients out of 17 with an epilepsy diagnosis. Of those treated, five patients received one AED, six patients received two AEDs, and three patients received three AEDs (Figure 2). The specific received AEDs are detailed in Figure 3.

Nineteen patients (59.4%) were treated with the mTOR inhibitor (everolimus), ten of whom were treated for 5 years; three patients were treated for 4 years; four patients for 3 years; one patient for 2 years; and two patients for only 1 year at the time of our study. The average duration of treatment with everolimus in our cohort was 3.67 years, while the average age at which patients started the treatment was 35.86 years.

In our study group, 21 of the 32 patients wanted to receive the mTOR inhibitor, and had at least one inclusion criterion and no exclusion criterion according to our national protocol. The 21 patients started receiving everolimus; 3 had side effects at 6 to 12 months after treatment initiation (one developed active tuberculosis and completely stopped the treatment after 6 months), while 2 patients developed severe neutropenia in the first 6 months after treatment initiation and had a dose reduction. Of these two patients, one developed a severe dental infection at 12 months after treatment initiation and completely stopped the treatment, while the other patient stabilized after the dose reduction, and continues everolimus to date. Thus, 19 out of 21 patients (90.48%) receiving the mTOR inhibitor were stable from a neurological and renal point of view (repeated clinical and imaging assessments) and continued to have a benefit from the mTOR inhibitor at an average of 3.67 years after initiation.

### 3.5. Association of Variables

We found that epilepsy was positively associated with intellectual impairment (*p* = 0.018, OR = 12.44, CI [1.32, 117.03]).

The statistical analysis further revealed a positive association between epilepsy and the presence of cortical tubers (*p* < 0.0010). Also, the positive association between epilepsy and SEN was significant (*p* = 0.036). However, only SEN observed in neuroimaging were found as a risk factor for intellectual impairment (*p* = 0.015, OR = 9.92, CI [1.60, 61.60]).

Male patients showed more dermatological manifestations than females (in particular, café-au-lait patches were found more frequently in male patients) but without reaching a statistical significance.

Café-au-lait patches were found in 30% of males versus 5% of females, but the difference did not reach statistical significance (*p* = 0.53), indicating a trend that should be taken into consideration in further studies.

The Charlson comorbidity index scores were significantly higher for female patients in comparison with males, with mean score 4.95 versus 3.54. The exact significance for the Mann–Whitney U Test was *p* = 0.033, indicating a higher burden of disease in females.

**Figure 4:**
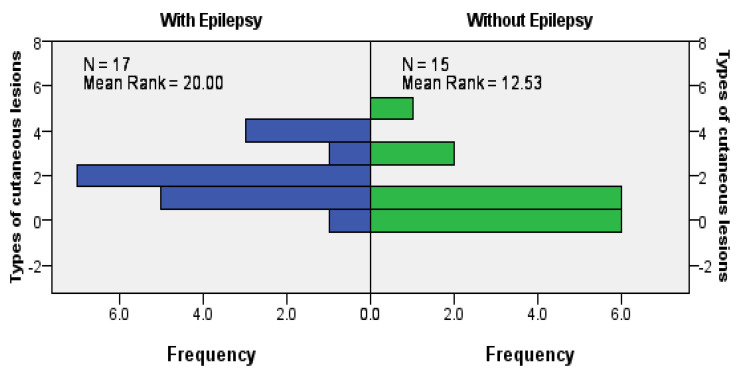
Association between epilepsy and total dermatological involvement (the number of different types of cutaneous lesions for each patient).

When looking at the relationships between dermatological manifestation and neurological manifestations or brain lesions, we found a statistically significant positive association between epilepsy and the presence of cutaneous angiofibromas (*p* = 0.027), more clearly seen in female patients (*p* = 0.020). We also observed a positive association between epilepsy and total dermatological involvement (exact significance for the Mann–Whitney U Test was *p* = 0.024; Figure 4).

## 4. Discussion

According to the largest worldwide study of tuberous sclerosis—the TOSCA Study [4,5,6]—gender distribution slightly favors females; our group confirmed this trend. However, the mean age at diagnosis of patients from Romania was significantly higher (7 vs. 11 years in our patients), with no differences observed between genders in comparison with the study by Nabbout et al. [4]. Due to costs, only two of our patients underwent genetical testing, whereas more than 45% of patients worldwide with clinical criteria for TSC had been tested. In our study, a trend towards an earlier diagnosis in females was observed, not previously mentioned in other studies [4,14]. This indicates that efforts of health providers in Romania should increase to provide an earlier diagnosis of the affection, so as to ensure earlier treatment and a higher quality of life in adulthood.

The most important neurological components of TSC are specific brain lesions (cortical dysplasia (including tubers and white matter migration lines), SEN, and SEGA), epilepsy, and TAND [1,2,4]. The above-mentioned lesions can be observed in 80–90% of patients and are frequently associated with epilepsy, which also affects up to 85% of patients and represents an important cause of disability and mortality in TSC [1,4]. In our group, only 22 of the 32 patients managed to undergo neuroimaging and the rates of detection for each type of brain lesion were lower than those in the existing literature, possibly due to technical issues and to lack of experience of the interpreting radiologist, especially when imaging was performed in smaller cities or rural areas. Among the patients with neuroimaging results available, 21 (95.5%) had specific TSC brain lesions and 17 (77.3%) had a diagnosis of epilepsy. As expected, epileptic seizures as the presenting symptom that ultimately led to TSC diagnosis were reported by 16 of the 17 patients. However, only 53.1% of our cohort had an epilepsy diagnosis, significantly smaller than the indicated rates in the available literature. Intellectual disability of various degrees was reported in up to 50% of patients with TSC [1,2,4]. Our study identified a smaller rate (28.1%) among our patients, possibly due to lack of appropriate neuropsychological testing. We also found a significant correlation between the use of antiepileptic drugs and cognitive impairment, expressing the deep impact that epileptic seizures, controlled or not, have on the normal psychomotor development. This underlies the importance of a comprehensive evaluation as soon as a TSC diagnosis is suspected, especially in a patient with seizures, as mild intellectual impairment can easily be overlooked and can be aggravated in the absence of any therapeutic intervention.

Antiepileptic treatment for epilepsy associated with TSC in adults respects the general guidelines and depends on patient and comorbidities. Unfortunately, a significant percentage eventually become drug-resistant and require a ketogenic diet, epilepsy surgery, vagus nerve stimulation, or other interventions to obtain a better control of the seizures [1,2,4]. Since the beginning of the national program for tuberous sclerosis patients in Romania in 2014, 21 out of the 32 patients in our group opted for the mTOR inhibitor treatment and met the inclusion criteria. At the time of data collection, around 60% (19 out of 32) of our patients benefited from treatment with the mTOR inhibitor (everolimus), more than half of whom had been treated for 5 years at the time of inclusion in the study. Thus, the general retention rate for everolimus was 90.48% (19 out of 21 patients), indicating that it is a well-tolerated treatment with a low incidence of severe side effects leading to treatment cessation, especially in the first year after initiation. Dose adjustments should be individualized, and special attention should be paid to prevention and management of acute infections. In our study, 11.8% (2 out of 17) patients with epilepsy did not require AEDs, obtaining a satisfactory control of seizures from treatment alone with everolimus. These patients only had cortical tubers in their neuroimaging. This finding supports previous studies on mTOR inhibitors’ efficacy on seizure frequency in patients without SEGA [11,12].

Dermatological manifestations of TSC usually occur in more than 90% of patients, and the specific lesions make up almost half of the major and minor diagnostic clinical criteria, some distinguishable from early infancy [8,14,15]. Considering that about 80% of our group had documented cutaneous and oral lesions relevant for TSC diagnosis and that hypomelanotic macules were recognized in around 15% of patients, educational interventions should be enforced to assist clinicians in easier recognition of these manifestations. The most frequently observed lesions in our patients were facial angiofibroma, which is expected to be easily observable in an adult population [8,16]. Curiously, a trend of more frequent café-au-lait lesions in males with TSC was observed, not previously mentioned in other studies [14,15,16].

In our cohort, we found a statistically significant association between cutaneous angiofibroma and epilepsy, more clearly in female patients, confirming the validity to this day of the triad proposed by Vogt in 1908 for the diagnosis of TSC. We also found a significant relationship between epilepsy diagnosis and total dermatological involvement.

The Charlson comorbidity index (CCI) has been developed to categorize the comorbidities of patients and predict mortality rates [17,18]. An interesting relationship we found was that females had a significantly higher CCI score, which indicates a higher burden of disease in females with TSC with possible consequences regarding survival. However, a recent study by Chu et al. [19] did not find any statistically significant differences in survival time and mean age of death between genders, but treatment with an mTOR inhibitor was given to only 16.5% of patients. More longitudinal studies are needed, especially after the increase in availability of mTOR inhibitors, to assess gender differences in burden of illness and mortality of TSC patients.

## 5. Conclusions

Tuberous sclerosis complex requires a team effort for appropriate management considering the multiple comorbidities and high percentage of patients with intellectual impairment and epilepsy. Cortical tubers in neuroimaging seem to be associated with the diagnosis of epilepsy, while subependymal nodules represent a risk factor for intellectual impairment. Dermatological lesions, although widely described in the literature, were insufficiently recognized in our cohort and educational measures are recommended. In our group, males showed more dermatological lesions, especially café -au-lait patches, but without reaching a statistical significance. Interestingly, we found a statistically significant positive association between epilepsy and the presence of cutaneous angiofibromas, as well as total dermatological involvement. Considering all comorbidities and their impact on the quality of life, females had significantly higher Charlson comorbidity index scores, indicating a higher burden of disease. Everolimus seemed to be a well-tolerated treatment and showed promising results in controlling epileptic seizures alone in two patients. More studies, with the inclusion of a larger number of patients, are needed to confirm these results.

## 6. Limitations

One of the major limitations of our study was the small number of included patients. Unfortunately, there is no national registry of tuberous sclerosis patients in Romania, but efforts are made towards this goal. Thus, only the 32 patients who are monitored in our hospital (the largest number at one single center in our country) could finally be included in our study. Another limitation was the short duration of treatment with everolimus, since the national program making this medication available to the Romanian population was started in 2014. The patients are continuously observed, and we hope to have more interesting data in the following years. A third limitation was the absence of genetic mutation determinations, because the genetic testing costs are still high in Romania and must be supported solely by the individual patient or family, thus rarely available.

## Data Availability

Data available on request due to privacy restrictions.
